# Supplementary material for: Transcriptomic Insights into Mechanisms of Early Seed Maturation in the Garden Pea (Pisum sativum L.)
Source: Cells. 2020 Mar 23;9(3):779. doi: 10.3390/cells9030779 (PMC7140803; doi:10.3390/cells9030779)

Input data distribution

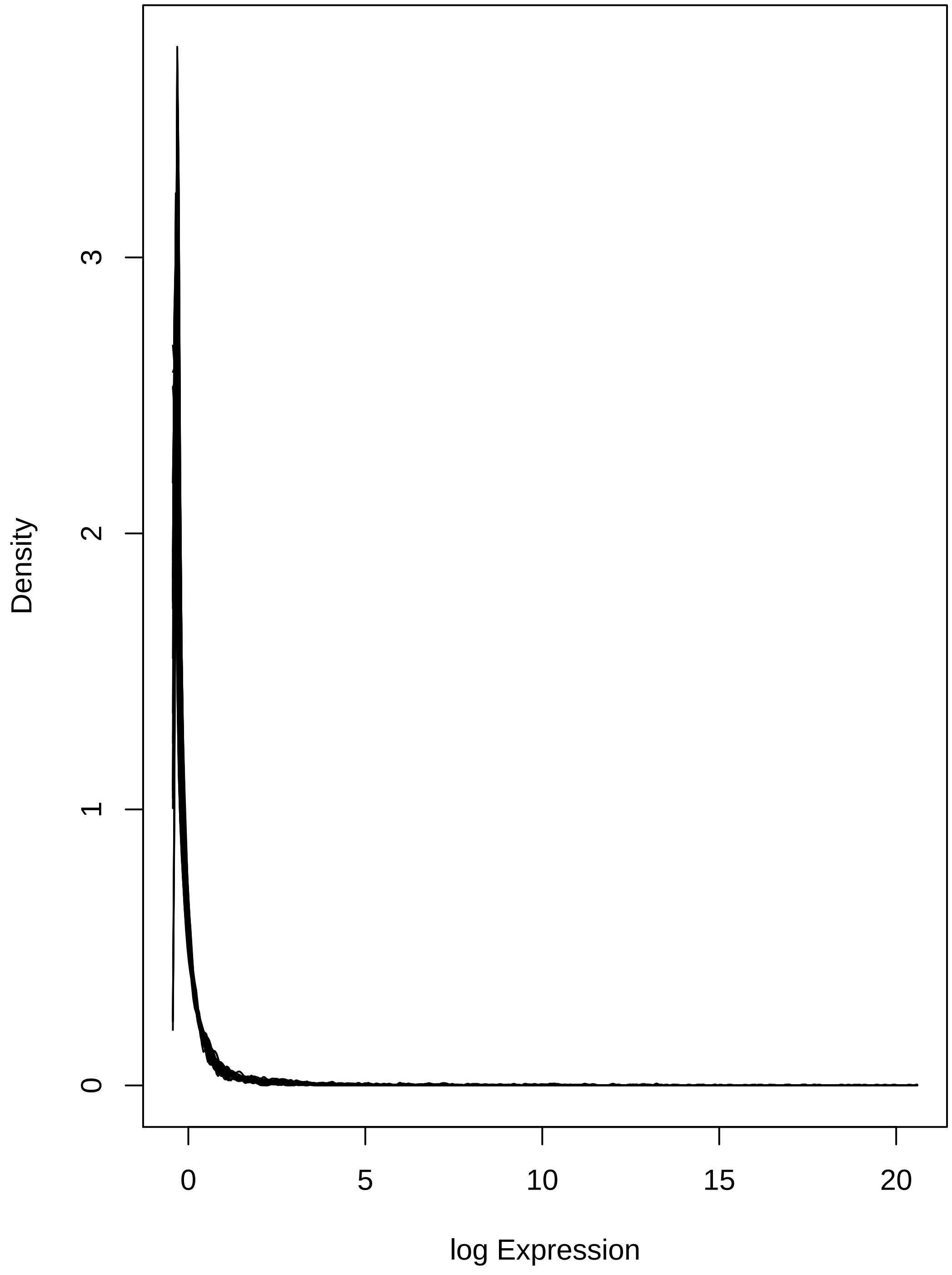

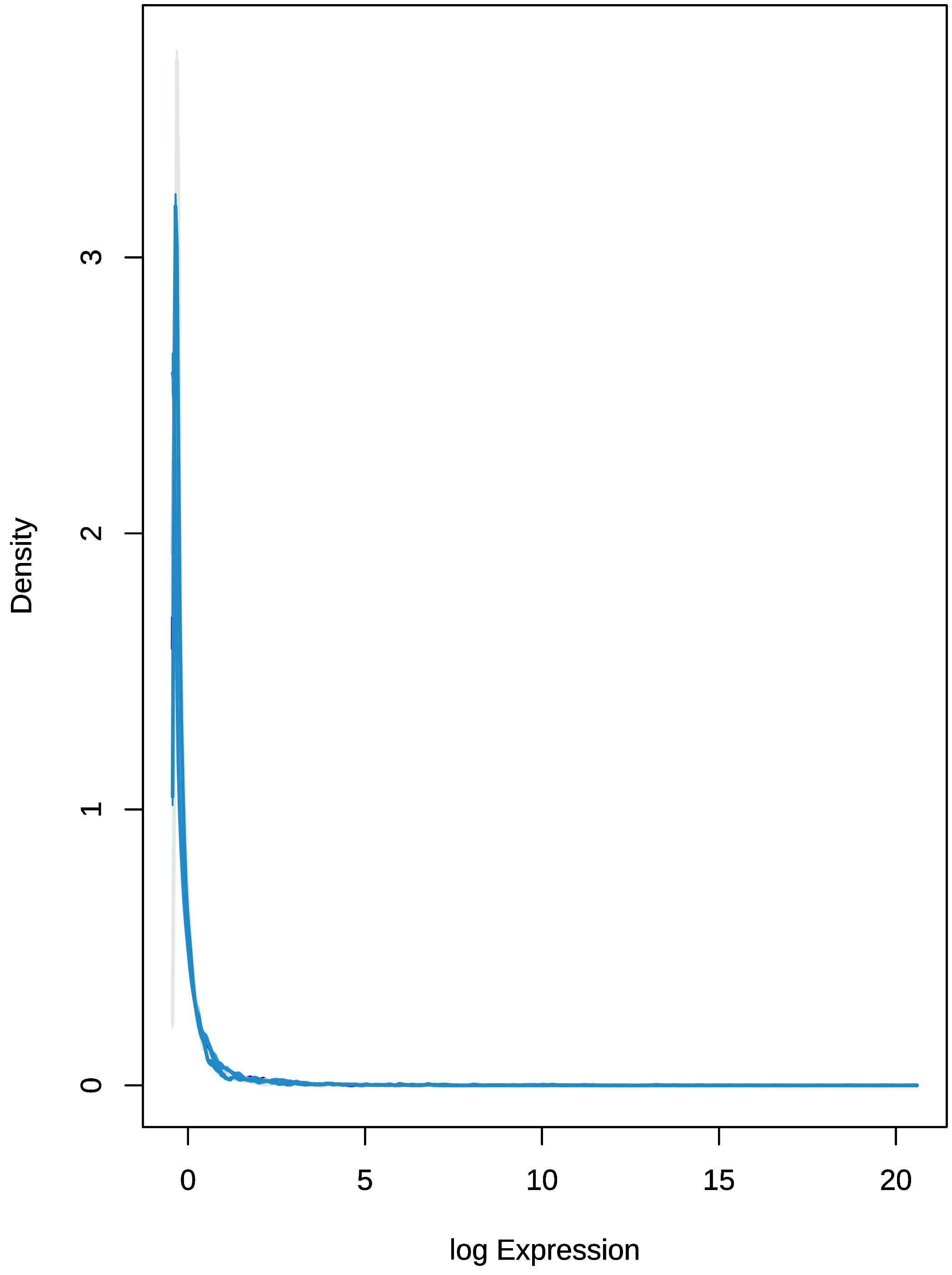

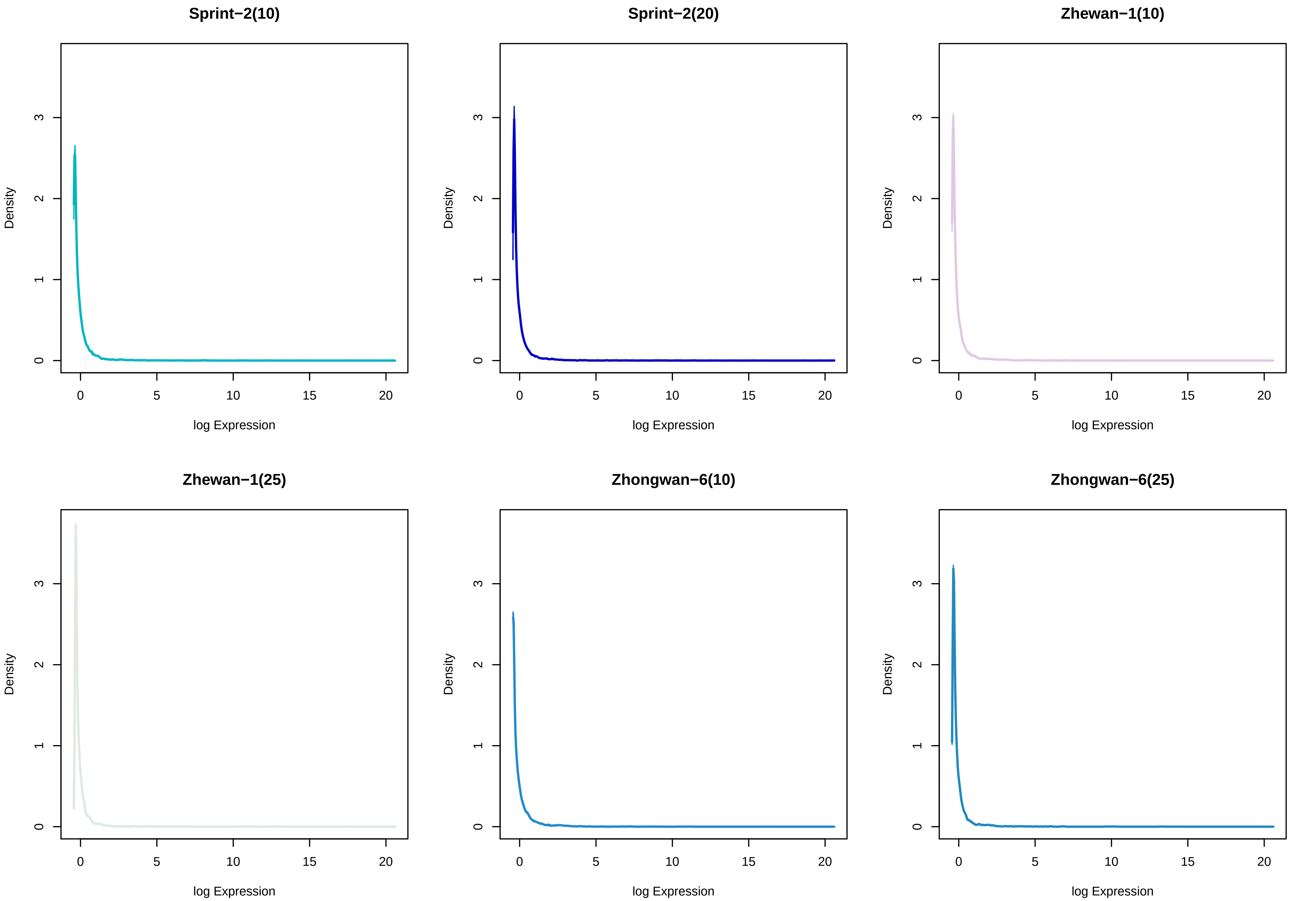

Sprint−2(10)
Sprint−2(20)
Zhewan−1(10)
Zhewan−1(25)
Zhongwan−6(10)
Zhongwan−6(25)
Density
log Expression
0
1
2
3
0
5
10
15
20

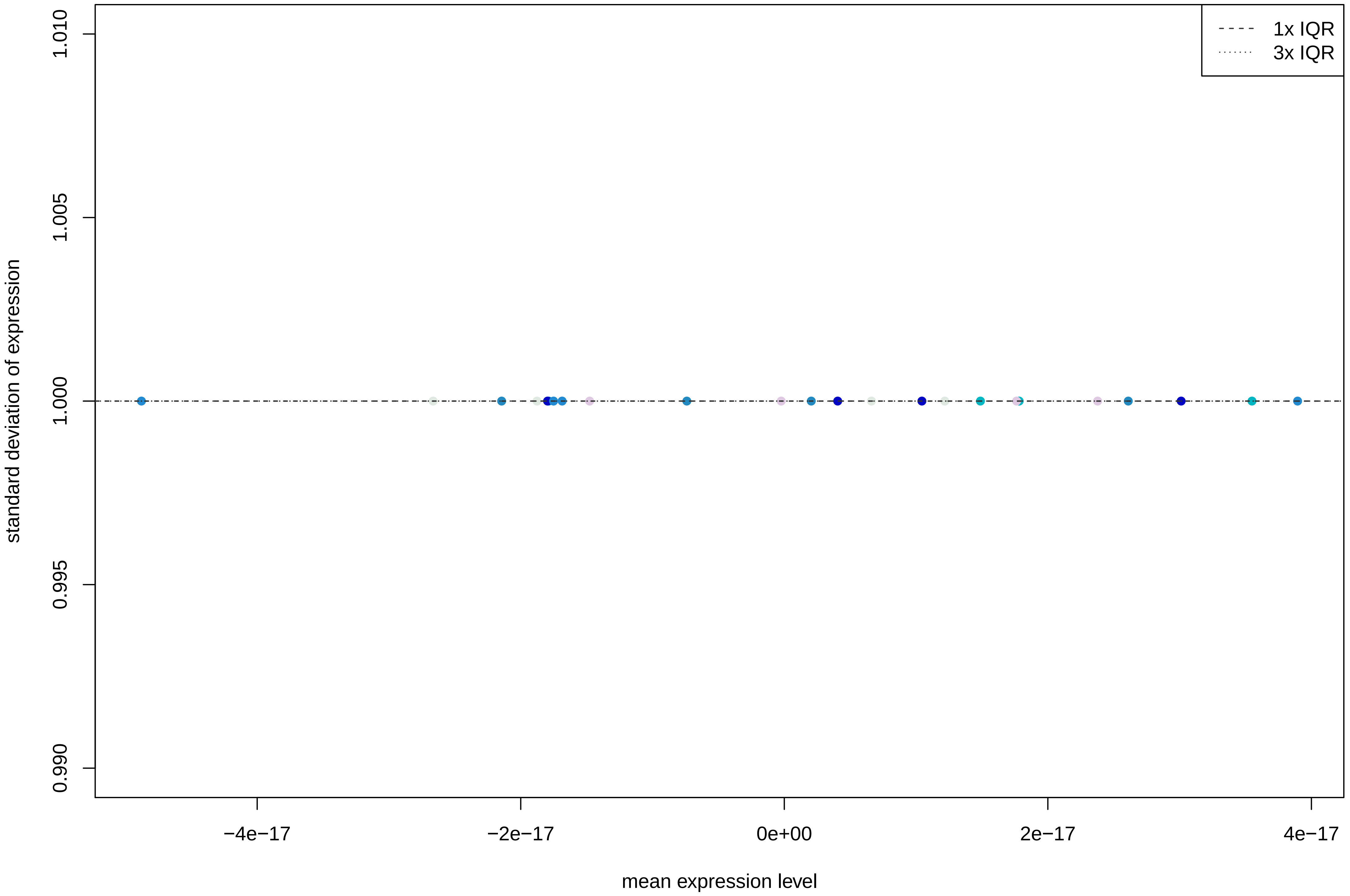
1x IQR
3x IQR
standard deviation of expression
1.010
1.005
1.000
0.995
0.990
−4e−17
−2e−17
0e+00
2e−17
4e−17
mean expression level

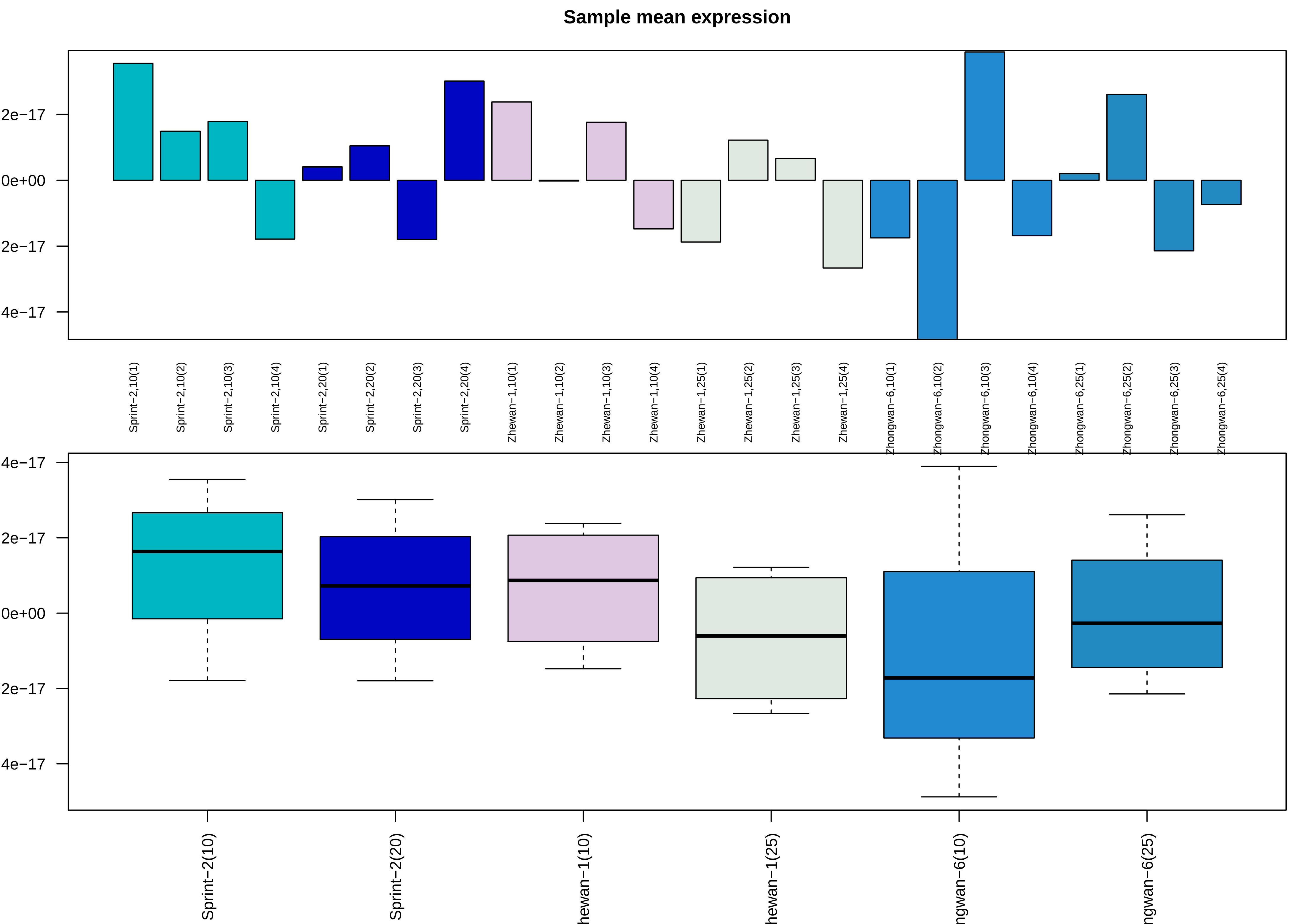
Sample mean expression
2e−17
0e+00
2e−17
4e−17
Sprint−2,10(1)
Sprint−2,10(2)
Sprint−2,10(3)
Sprint−2,10(4)
Sprint−2,20(1)
Sprint−2,20(2)
Sprint−2,20(3)
Sprint−2,20(4)
Zhewan−1,10(1)
Zhewan−1,10(2)
Zhewan−1,10(3)
Zhewan−1,10(4)
Zhewan−1,25(1)
Zhewan−1,25(2)
Zhewan−1,25(3)
Zhewan−1,25(4)
Zhongwan−6,10(1)
Zhongwan−6,10(2)
Zhongwan−6,10(3)
Zhongwan−6,10(4)
Zhongwan−6,25(1)
Zhongwan−6,25(2)
Zhongwan−6,25(3)
Zhongwan−6,25(4)
4e−17
2e−17
0e+00
2e−17
4e−17
Sprint−2(10)
Sprint−2(20)
hewan−1(10)
hewan−1(25)
ngwan−6(10)
ngwan−6(25)

Sample expression standard deviation

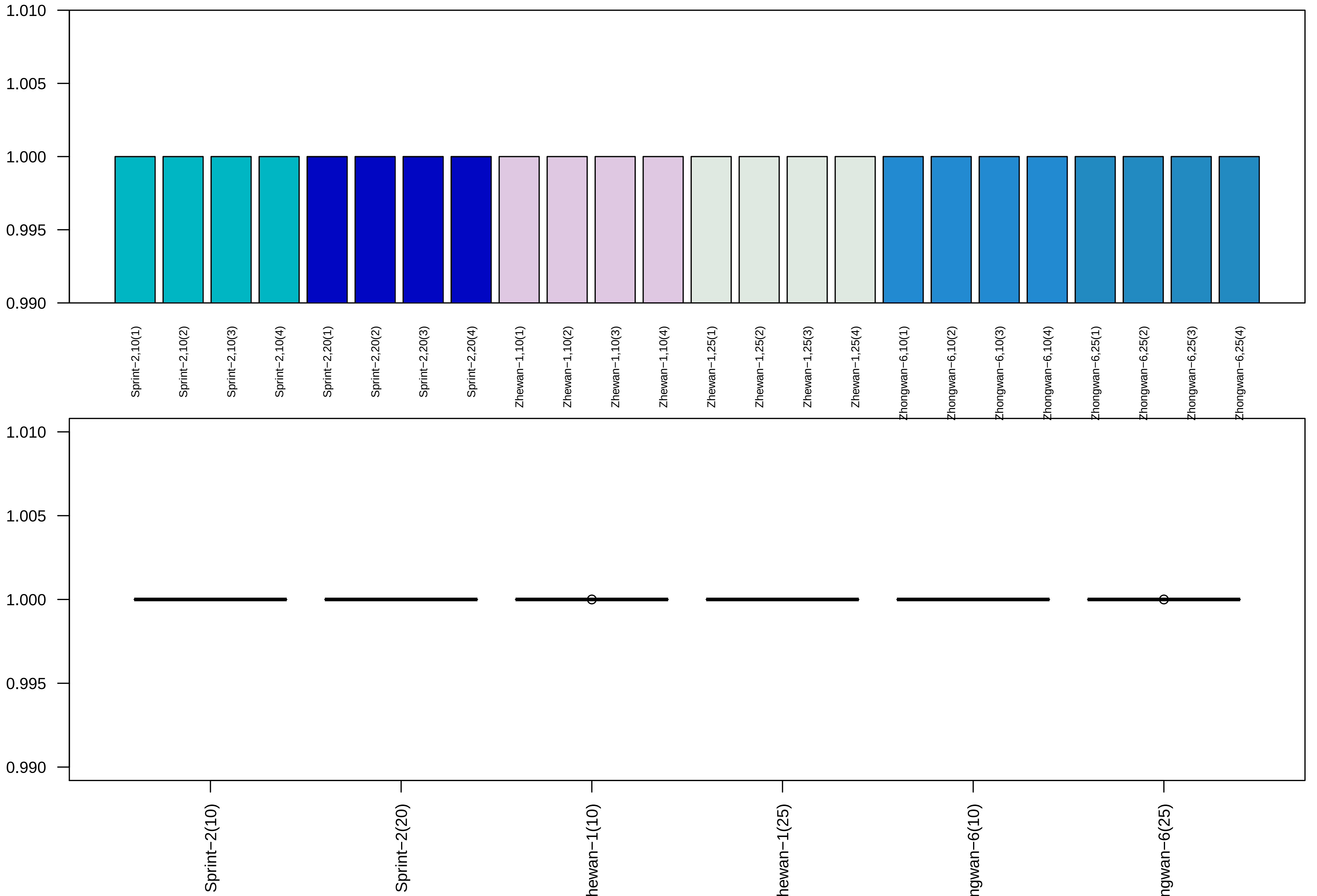

Supplement: Supplementary file 1 [file cells-09-00779-s001.zip › Supplementary materials/FileS4.zip!/FileS3/TFs - Results/Data Overview/Data Distribution.pdf]
